# Role of emergency VATS in blunt chest trauma patients

**DOI:** 10.1186/1749-8090-8-S1-O73

**Published:** 2013-09-11

**Authors:** A Gabal, M Alghorori

**Affiliations:** 1 Thoracic Surgery Unit, Department of Surgery, King Saud Hospital, Al-Qaseem, Kingdom of Saudi Arabia

## Background

Thoracic injury directly accounts for 25% of the deaths due to trauma, and is a contributing factor in another 25% of trauma related deaths. As the role of thoracoscopy continues to expand in the practice of thoracic surgery, its role in the management of the trauma patients is also expanding. Objective of the study was to determine the role of emergency VATS in the management of blunt chest trauma patients.

## Methods

From Jan. 2009 to Dec. 2012, 80 blunt chest trauma patients underwent emergency video-thoracoscopy 10 of them was done under sedation with local anesthesia. The patients’ charts were reviewed for demographic data, mechanism of injury, procedures performed, outcome, and length of hospital stay.

## Results

All VAT procedures were performed within 24 hours except for 3 cases with retained haemothorax as the procedure was repeated again at day 7. 50 (62.5%)patients had haemothorax not more than 800ml, the cause of haemothorax varied from a parenchymal laceration, and intercostal muscle bleeding in most cases, and in some patients was due to (diaphragmatic injuries – pericardial tear – and intercostal artery lesion) , in 4 patients the pathology was not clear. 15 patients (18.75%)had pneumothorax , simple rib fractures was detected among 10 of them , the procedure did not identify any parenchymal or vascular lesions. In the remaining 15 patients (18.75%), the diagnosis was haemopneumothorax (5 with flail chest and 10 with multiple rib fracture) . Seven patients required conversion to an open thoracotomy. ICT stayed in our patients from 2 to 15 days (Average 5.91±2.6). The average length of stay in the hospital after VATS was 9.1±2.73 days (vary from 4 to 21 days). There was no mortality.

## Conclusions

In our study we found that VATS in haemodynamically stable patients with blunt chest trauma is safe and effective, and it can be performed with some diagnostic benefits in sedated patients avoiding the hazards of general anesthesia.

